# Expression of Estrogen Sulfotransferase 1E1 and Steroid Sulfatase in Breast Cancer: A Immunohistochemical Study

**DOI:** 10.4137/bcbcr.s2012

**Published:** 2009-03-20

**Authors:** D. Poisson Paré, D. Song, V. Luu-The, B. Han, S. Li, G. Liu, F Labrie, G. Pelletier

**Affiliations:** 1 Molecular Endocrinology and Oncology Research Center, Laval University Hospital Research Center, 2705 Laurier blvd, Quebec City, Qc, Canada, G1V 4G2; 2 First Hospital of Jilin University, Changchun, China

**Keywords:** breast cancer, EST1E1, STS, immunohistochemistry

## Abstract

It is known that the steroid sulfatase (STS) and the estrogen sulfotransferase (EST1E1) are commonly expressed in human breast carcinomas. STS and EST1E1 combined action could maintain the equilibrium between sulfated (inactive) and unconjugated (active) estrogens, which might have effects on development of hormone dependent breast cancer.

We studied the expression of the STS and EST1E1 in 88 breast carcinomas and 57 adjacent non-malignant tissues by immunohistochemistry. The results were correlated with the tumor expression of estrogen receptor α (ER-α) and β (ER-β), progesterone receptor A (PR-A) and B (PR-B) and the proliferation marker CDC47, the tumoral type and stage and the age at surgery.

STS expression was higher in carcinoma specimens than in adjacent normal tissues, although not to a significant level (p = 0.064) and it was positively associated with CDC47 expression (p < 0.05). These observations support the hypothesis that STS is overexpressed in breast cancer and associated with a worse prognosis.

EST1E1 was observed for the first time in the nuclei of epithelial and tumoral cells. Tumor expression of EST1E1 was positively correlated with ER-β (p < 0.01) and PR-B (p < 0.05), two steroid receptors already associated with an improve prognosis for breast cancer.

Controlling the STS overexpression in carcinomas could be a way to inhibit cancer growth. The significance of the association between EST1E1 and ER-β or PR-B should be further studied since these two receptors are transcription activators and may regulate the expression of protective enzymes like EST1E1.

## Background

It is well documented that increased exposure to estradiol (E2), the most potent estrogen, is an important risk factor for the development of breast cancer. Approximately 95% of breast cancers are estrogen-dependent in their early stage, whether in premenopausal or postmenopausal women.^1^ However, two-thirds of breast cancers are diagnosed after menopause, when the ovarian exhaustion leads to a dramatic decrease of E2 serum levels. Interestingly, it was determined that the intratumoral E2 concentration in postmenopausal patients is however maintained at a level similar to that found in premenopausal patients.^2^ This observation suggests an intratumoral biosynthesis of E2.

One important feature responsible for the tumoral production of E2 is the desulfation of the inactive estrone sulfate (E1-S) by the steroid sulfatase [steryl sulfatase, EC 3.1.6.2 (STS)], an enzyme ubiquitously expressed in many organs and particularly in breast carcinoma tissue.^3^ Estrone (E1) is synthesized in peripheral tissues following the aromatization of androstenedione (Δ4-dione) and subsequently sulfated.^4^ E1-S is the most abundant circulating estrogen in postmenopausal women and its levels are 7 to 11 times higher in tumor tissues than in circulation.^2^ Once desulfated, E1 is subsequently reduced into E2 by the 17β-hydroxysteroid dehydrogenases (17β-HSD) types 1, 7 and 12.^5^ The presence of the 17β-HSD enzymes in breast tumor has been previously shown, supporting the potential role of the STS pathway.^5^–^8^

STS can also convert the sulfated compound dehydroepiandrosterone (DHEA-S) in unconjugated DHEA, an inactive androgen that can be transformed in Δ4-dione, the main estrogen precursor.^4^ STS can then affect E2 production at two different levels: direct transformation of conjugated estrogens in active estrogens (E1-S to E1, E2-S to E2) and increase of Δ4-dione, which can be subsequently aromatized into E1. Previously, the expression of STS has been reported in a large proportion of breast cancer cases.^9^–^13^ Furthermore, high levels of STS were linked to poor prognosis and increased risks of recurrence.^12^,^14^,^15^

In opposition to the STS action, E1 and E2 can be transformed into inactive E1-S or E2-S by sulfotransferases (SULT). So far, several SULT enzymes have been identified, including SULT1A1, 1A2, 1A3 and 1E1. However, the 1E1 enzymes, also known as estrogen sulfotransferase (EST1E1) [EC 2.8.2.4] is the one which have the highest affinity for estrogens.^16^ The estrogen sulfonation is an important feature to protect peripheral tissues from possible excessive estrogenic effect since the addition of the sulfonate group prevents the binding of the estrogen to its receptor.^17^ Expression of EST1E1 has been found in normal human mammary epithelial cells^18^,^19^ and in breast cancer tissue^12^,^20^ where it has been associated with an improve prognosis.

STS and EST1E1 combined action could maintain the equilibrium between sulfated and unconjugated estrogens, which might have effects on genesis and development of hormone dependent breast cancer. The main purpose of this study is to provide more information about the involvement of these enzymes in breast cancer. To accomplish this, we developed specific antibodies against STS and EST1E1 that we used to analyze the expression of these enzymes in human carcinomas and adjacent normal breast tissues by immunohistochemical localization studies. To the best of our knowledge, no studies have already compared the expression of EST1E1 between breast carcinomas and adjacent normal tissues from the same patients, although such an analysis has been already performed for the STS.^15^,^21^,^22^

The expression of STS and EST1E1 evaluated in breast carcinomas were also correlated with clinicopathological parameters such as estrogen receptor α (ER-α), estrogen receptor β(ER-β), progesterone receptor A (PR-A), progesterone receptor B (PR-B) and cell division control protein 47 (CDC47) expression as well as type and stage of the tumors and age and estimated menopausal status of the patients at surgery.

## Materials and Methods

### Patients

This study was approved by the institutional review board at the First Teaching Hospital of Jilin University and Tumor Hospital of Jilin Province, Changchun, China. This study was also approved by the ethical committee of the “Centre hospitalier de l’université Laval” (CHUL) Research Centre. Eighty-height women with primary breast cancer agreed to participate in this research project and gave us an informed consent. Patient characteristics are summarized in Table 1. Menopausal status was unknown for most of the patients so it was assumed that 50-year-old patients and older were usually menopaused. All patients underwent modified radical mastectomy without any preoperative therapy at the First Hospital of Jilin University and Tumor Hospital of Jilin Province (China) during the year 2004. The samples of tumors and adjacent non-neoplastic tissues taken out at more than 5 cm from the tumors were collected at surgery from patients with a mean age of 50.9 years (range 32–73). All samples were fixed in 10% formol in 0.2 M phosphate buffer (pH 7.4) for 24 hours. They were then dehydrated through increasing concentrations of ethanol and toluene and finally embedded in paraffin.

Among the 88 adjacent tissues taken out at more than 5 cm of the tumors, 17 samples contained only fat without acini, 12 samples showed inflammation signs and 2 contained only skin with sebaceous glands. These tissues were excluded while the 57 other adjacent normal tissues were included in our analysis.

### Immunohistochemistry

The breast carcinomas and adjacent breast paraffin sections (5 μm of thickness) were first deparaffinized in toluene, hydrated and then treated with 3% H_2_O_2_ in methanol for 20 minutes in order to eliminate the endogenous peroxidase.

For CDC47 and hormone receptor localization, these steps were followed by a treatment for antigen retrieval, as previously described.^23^ Tissues were then incubated with commercial antibodies against CDC47, ER-α, ER-β, PR-A or PR-B (Table 2). Commercial detection system kit (Covance Research Products, Inc., Dedham, Massachusetts) using the streptavidin-biotin amplification method was used afterward for the localization of CDC47 and the receptor. Finally, the antigen-antibody complex was visualized with a solution of PBS 1X containing 20 mg/100 ml of 3,3-diaminobenzidine and 3% H_2_O_2_. Nuclei were counterstained with hematoxylin.

**Figure 1.**
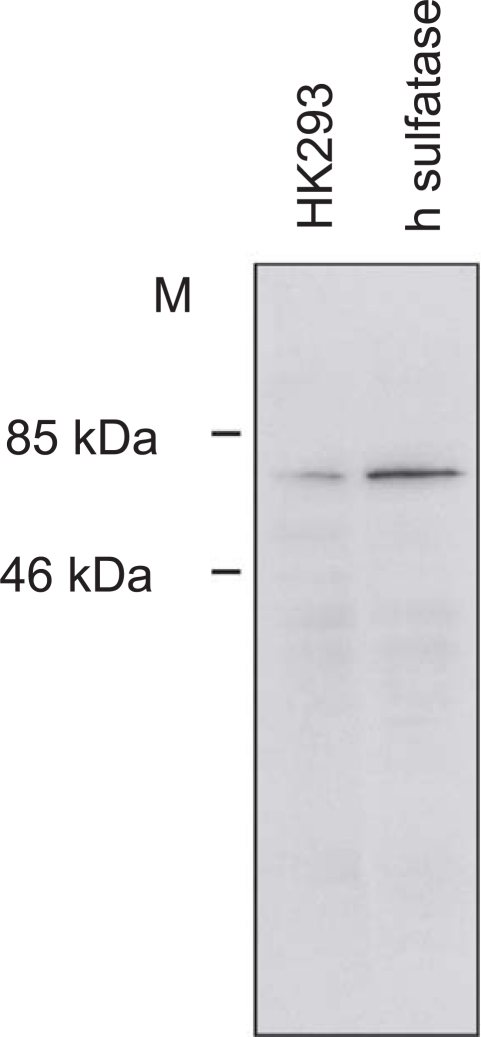
Validation of the STS antibody specificity. Western blot analysis of proteins from untransfected or transfected HK293 cells stably expressing human STS (Mw: 62 kDa). The antiserum specifically reacts with the overexpressed enzyme.

**Figure 2.**
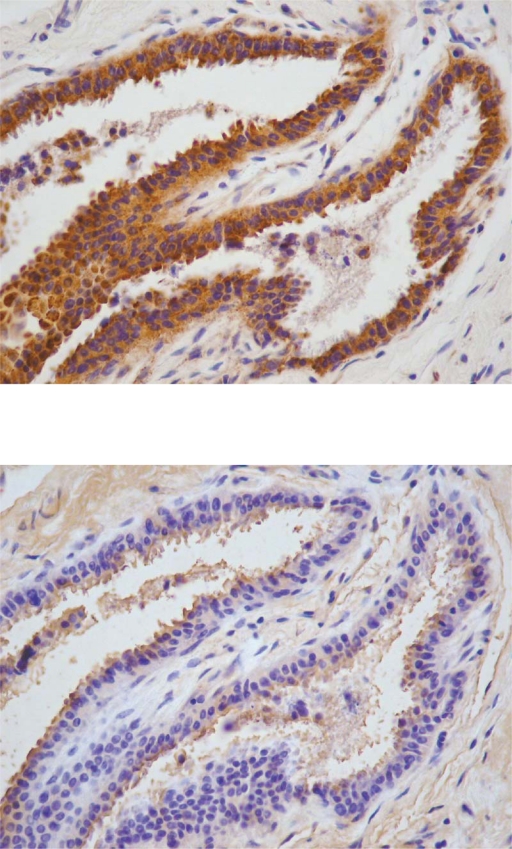
Immunostaining for STS in human breast carcinoma (dilution 1:100). Positive reaction is observed in the cytoplasm of cancerous cells C) 2A). Immunoabsorption of the antiserum with an excess of antigen (10^−6^ M) has completely prevented immunostaining in an adjacent control section 2B). (400X).

The specificity of the antiserum against EST1E1 used in this study has been described previously.^24^ The antibody against STS was raised in New Zealand rabbits using the peptide sequence corresponding to amino acids 38–165 of the human sulfatase. It was made by exactly the same method previously used for the development of EST1E1 antibody.^24^ The antibody’s specificity was verified by Western Blot (Fig. 1) and by immunoabsorption immunohistochemical studies (Fig. 2). STS and EST1E1 antibodies were diluted 1:250 and 1:100 respectively in this study.

For STS and EST1E1, no antigen retrieval was performed. Furthermore, goat anti-rabbit antibodies linked to the horseradish peroxidase were used instead of the commercial kit for STS and EST1E1 localization. The antigen-antibody complex was visualized with a solution of PBS 1X containing 20 mg/100 ml of 3,3-diaminobenzidine and 3% H_2_O_2_. Nuclei were counterstained with hematoxylin.

For all the immunohistochemical experiments, negative controls were performed on adjacent sections by using commercial normal rabbit serum or normal mouse serum instead of the primary antibodies. These sections were negative (results not shown).

### Scoring of immunoreactivity

The data were generated after fully reviewing the complete sections of each human carcinoma and adjacent tissue. According to previous studies,^7^,^12^ two researchers independently classified the STS and EST1E1 in three groups: <1% positive cells, no immunoreactivity; l%–50% positive cells, 1+; more than 50% positive cells, 2+. Scoring of ER-α, ER-β, PR-A, PR-B and CDC47 was performed using the same classification method. The intensity of labeling was not considered due to variations in the background staining between sections.

Inter-observer differences between 1+ and 2+ occurred in 17.9% of STS cases and in 7.1% of EST1E1 cases. This discrepancy was mainly due to the fact that the real percentage of positive cells was often located at the interstice of these two groups (around 50%). Inter-observer differences between negative and 1+ or between negative and 2+ occurred in less than 5% of STS and EST1E1 cases. These differences appeared only when the staining was very weak or the background very high. Duplicates of immunohistochemistry were performed for these cases. All the cases with different estimations between observers were discussed and reevaluated until consensus.

### Statistical analyses

The Chi-square (χ2) test or the Fisher’s exact test were used to compare the enzyme expression between the carcinomas and the normal tissues and to assess associations between categorical variables. The association between enzyme expression and age (continuous variable) was analyzed using Spearman correlation coefficients. All analyses were performed using SPSS software (Version 16.0).

## Results

### Expression of STS and EST1E1

**Figure 3.**
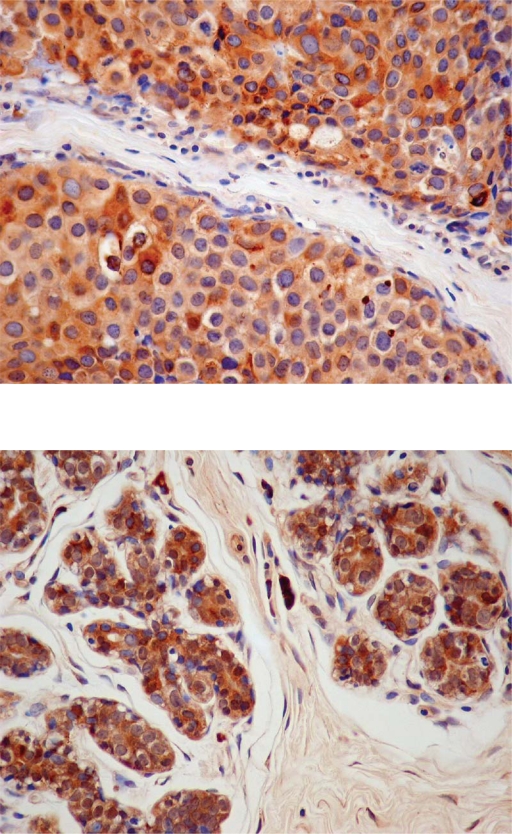
Localization of the enzymes in infiltrating ductal carcinoma (3A and 4A) and in adjacent normal breast tissue (3B and 4B). X400. 3) Immunostaining for STS is observed in the cytoplasm of cancerous cells C) (3A) and in the cytoplasm of epithelial cells E) 3B). 4) Immunostaining for EST1E1 is detected in the cytoplasm and nuclei of cancerous cells C) 4A) and in both the cytoplasm and nuclei of epithelial E) and stromal cells (S) 4B).

Immunostaining for STS was almost exclusively cytoplasmic (Fig. 3). It was seen in the cytoplasm of malignant cells in all the cancer cases: 31 invasive carcinomas (35.2%) were 1+ (1%–50% cells stained) and 57 (64.8%) were 2+ (more than 50% stained cells). In non-malignant adjacent tissues, STS staining was seen in the epithelial cells of both acini and ducts in 55 out of 57 samples (96.5%): 28 (49.1%) were 1+ (1%–50% cells stained) and 27 (47.4%) were 2+ (more than 50% stained cells). The percentage of stained cells was higher in the invasive carcinomas than in the normal adjacent tissues, although not to a significant level (p = 0.064) (Fig. 5A).

**Figure 4.**
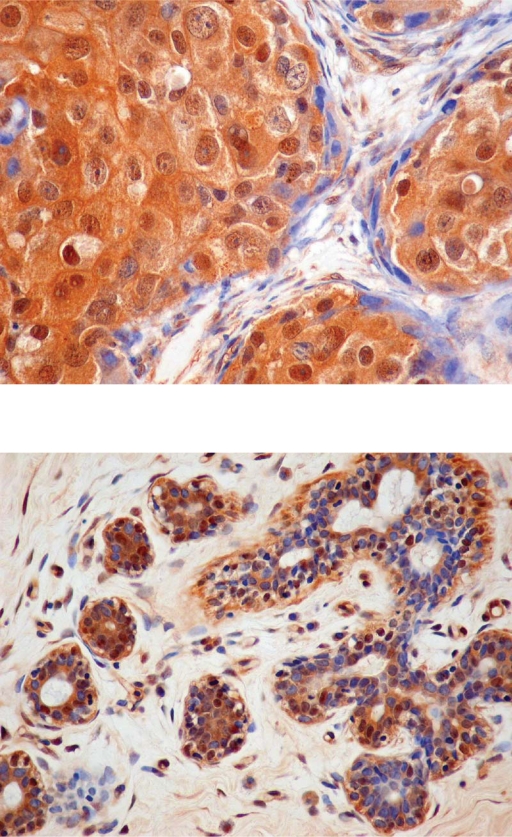
Localization of the enzymes in infiltrating ductal carcinoma (3A and 4A) and in adjacent normal breast tissue (3B and 4B). X400. 3) Immunostaining for STS is observed in the cytoplasm of cancerous cells C) 3A) and in the cytoplasm of epithelial cells E) 3B). 4) Immunostaining for EST1E1 is detected in the cytoplasm and nuclei of cancerous cells C) 4A) and in both the cytoplasm and nuclei of epithelial E) and stromal cells (S) 4B).

Cytoplasmic and nuclear staining for EST1E1 (>1% cells stained) was observed in 83 out of 88 invasive carcinomas (94.3%): 30 (34.1%) had between 1%–50% stained cells and 53 (60.2%) had more than 50% stained cells (Fig. 4).

**Figure 5.**
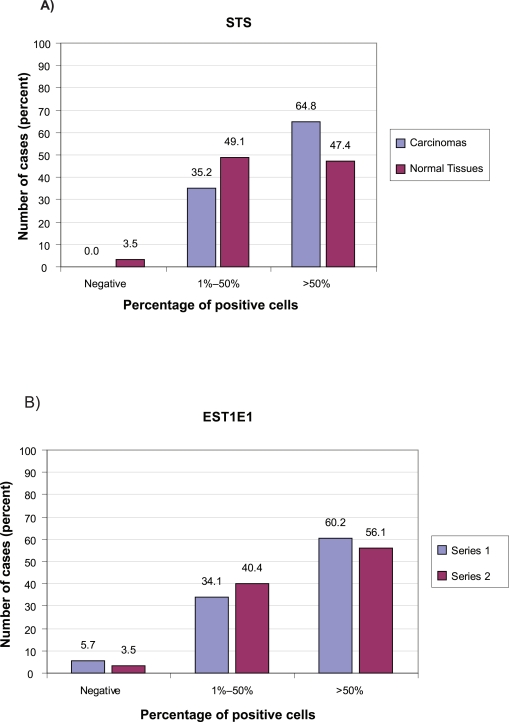
Comparison of the expression of STS **A)** and EST1E1 **B)** between cancer and normal adjacent tissues.

EST1E1 immunoreactivity was also found in 55 out of 57 adjacent normal tissue samples (96.5%). The staining was detected in the cytoplasm and nuclei of stromal cells and epithelial cells in both acini and ducts. Among the positive tissues, 23 (40.4%) had between 1%–50% stained cells and 32 (56.1%), had more than 50% stained cells. We could not measure any significant differences between the EST1E1 expression in invasive carcinomas and that observed in adjacent normal tissues (Fig. 5B).

### Expression of steroid receptors and CDC47

Immunostaining for ER-α, PR-B as well as CDC47 was exclusively observed in nuclei of epithelial cells in both non-tumoral adjacent tissues and carcinomas. Immunoreactivity for ER-β and PR-A was also found in normal epithelial cells and in tumoral cells but the staining was both nuclear and cytoplasmic.

As previously observed,^8^ the expression of CDC47 was significantly higher in tumors than in adjacent normal tissues (p < 0.001). The expression of ER-α was also significantly higher in the carcinomas than in the adjacent non-malignant breast tissues (p < 0.001). For all the other receptors, we could not measure any significant differences (Table 3).

### Correlations between STS, EST1E1, and the other parameters evaluated

Correlations between STS immunoreactivity and clinicopathological parameters are summarized in Table 4A. No significant correlations were observed between STS immunoreactivity and age, histological type and tumor stage. Furthermore, there was no correlation between STS expression and the expression of ER-α, ER-β, PR-A and PR-B. A positive association was however observed between STS and CDC47 (p < 0.05) and between STS and EST1E1 (p < 0.05).

As for STS, no significant correlation between EST1E1 immunoreactivity and clinicopathological parameters such as age, histological type and tumor stage could be observed (Table 4B). However, EST1E1 was positively correlated with CDC47 (p < 0.05), PR-B (p < 0.05) and particularly ER-β (p < 0.005). There was no correlation between EST1E1 expression and PR-A or ER-α.

## Discussion

The enzymes STS and EST1E1 are both involved in the intratumoral equilibrium between sulfated and unconjugated estrogens. STS generates bioactive E1 from E1-S while EST1E1 is responsible for the inverse reaction.

### STS

In this study, all the invasive carcinomas were positive for STS, which is in agreement with Tseng et al.^9^ who measured STS activity ranging from 0.2 to 4.6 nmol/mg tissue protein per hr in 66 breast carcinomas. The percentage of positive cases that we obtained (100%) is however higher than others previously found^10^ (89%);^11^ (88%);^13^ (59%);^12^ (74%). The inherent heterogeneity between the studied cohorts and the difference in the antibodies used may partly explain these differences. Furthermore, Yamamoto et al. have considered negative the cases with less than 10% of positive cells while we have considered negative the cases with less than 1% of positive cells.^13^

Immunohistochemical analyses have showed that STS expression is higher in carcinomas than in adjacent normal tissues (p = 0.064), although not to a significant level. This observation is consistent with the results reported by Utsumi et al.^22^ who found a higher STS mRNA level in breast cancers than in normal tissues adjacent to carcinomas. Our results are also in agreement with those of Tseng et al.^9^ and Chetrite et al.^21^ who reported that STS activity was higher in tumors than in normal adjacent tissues. CDC47 which is implicated in DNA replication has been used to study the cell proliferation in breast cancer section. Since STS was positively associated with CDC47 expression, it might be hypothesized that the overexpression of STS can lead to an increased estrogen-dependent proliferation of cancer.

As previously reported by other groups, we did not observe any associations between STS expression and age, tumor type or tumor stage.^12^,^13^ On the other hand, Myoshi et al. have previously reported a positive correlation between STS mRNA levels and tumor stage.^15^ As for other groups, we could not establish any link between STS and steroid receptor status.^10^–^13^

We could not compare the tumoral STS expression with tumor size or risk of recurrence because these informations were lacking for most of our cases. Other groups have found a significant positive correlation between STS levels and tumor size or a worsened prognosis.^12^,^14^,^15^ The association between STS and the mitotic marker CDC47 that we found support the relevance of these observations.

### EST1E1

In the present study, 94.3% of the carcinoma samples were positive for EST1E1. This percentage is higher than those reported by Hudelist et al. (79.4%) and Suzuki et al. (44.2%).^12^,^20^ The difference in antibodies used could probably explain this discrepancy. In fact, besides the cytoplasmic localization, we have observed a strong reaction in the nuclei of both normal and cancer samples. This is of interest because the observations previously reported showed only a cytoplasmic localization of immunoreactive EST1E1 in breast cells.^12^,^20^ Nuclear EST1E1 localization has been previously found in guinea pig adrenal cortex^25^ and in rat hepatocytes,^26^ supporting our observations that EST1E1 could have both nuclear and cytoplasmic subcellular localization. The high concentration of EST1E1 found in breast epithelial cell nuclei suggests that this enzyme play a role in modulating the ability of estrogens to regulate gene expression.

According to previous studies, high EST1E1 expression in cancer is associated with smaller tumors and a better prognosis.^12^,^20^ This seems logical since, as a consequence of the EST1E1 activity, cell proliferation should be inhibited following E2 inactivation. Our results are however in contradiction with these observations because EST1E1 was positively associated with CDC47 and STS expression in breast cancer (p < 0.05). It is important to keep in mind that immunohistochemical studies show only the expression of the protein and cannot give any information about the activity of the enzymes. It is possible that a high concentration of EST1E1 is produced to decrease the E2 concentration in high-grade tumors but these enzymes are not functional. Moreover, it cannot be excluded that other EST, such as EST1Al might be involved in E2 level regulation in breast cancer.^27^

A high positive association was found between EST1E1 and ER-β (p < 0.005). The involvement of ER-β in the development and progression of breast carcinoma cells is currently not well understood. Some groups have already observed a significant increase of EST1E1 when the tumors were ER+,^28^ or ER+/PR+.^9^,^29^ However, the antibodies used before the discovery of the ER-β subtype in 1996 by Kuiper et al.^30^ could probably recognize both ER-α and ER-β. There are studies indicating that ER-β expression is linked with smaller tumors showing lower histological grade and better disease-free and overall survival.^31^,^32^ Our observations suggest that ER-β can play its protective role by increasing the expression of enzymes which inactivate estrogens, such as EST1E1. This hypothesis should be further investigated.

Interestingly, there was a higher percentage of positive cells for EST1E1 among invasive carcinomas expressing high PR-B level than among invasive carcinomas expressing lower PR-B level (p < 0.05). Hopp et al. have already determine that PR-B act as a strong transcriptional activator while PR-A act as a transcriptional repressor and that a high PR-A:PR-B ratio is associated with a poorer diagnosis for breast cancer.^33^ According to our data, we can hypothesize that PR-B activates the transcription of protective enzymes in breast carcinomas, such as EST1E1. This receptor could be down-regulated in the worst cases of cancer, leading to a decrease of protective enzymes like ESS1E1. This hypothesis should be further investigated. Finally, PR-A and PR-B expression correlated with each other in breast cancers which also support Hopp et al. who have observed the same phenomenon.^33^

## Conclusions

In summary, we report that STS and EST1E1 are commonly expressed in human breast cancer. STS is overexpressed in carcinomas and associated with a high proliferation index. Controlling the STS overexpression could be a possible approach to decrease the hormone-dependent cancer growth.

We have observed for the first time a nuclear localization of EST1E1 in both normal and cancerous human mammary cells. Moreover, we have observed a positive association between two nuclear receptors, ER-β and PR-B, and the enzyme EST1E1. These new findings should lead to further investigations about the involvement of these receptors and their relation with protective enzyme in cancer growth and development.

## Figures and Tables

**Table 1.** Patient characteristics.

	**Cases (%)**
Age	
<50 years	45 (51.1)
≥50 years	42 (47.7)
Missing value	1 (1.1)
Tumor stage^a^	
I	7 (8.0)
II	68 (77.3)
III	12 (136)
Missing value	1 (1.1)
Histological type	
Infiltrating ductal	78 (88.6)
Infiltrating lobular	4 (4.5)
Others	5 (5.7)
Missing value	1 (1.1)

**Table 2.** List of primary antibodies.

**Antibody**	**Dilution**	**Source**	**Catalog no.**
ER-α	1:1000	Santa Cruz Biotechnology, Santa Cruz (CA)	SC-543
ER-β	1:100	Abcam (Cambridge, MA)	Ab288
PR-A	1:50	Medicorp (Montréal, Canada)	MS-197
PR-B	1:50	Medicorp (Montréal, Canada)	MS-192
CDC-47	1:500	Medicorp (Montréal, Canada)	MS-862

**Table 3.** Comparison of the expression of steroid receptors and CDC47 between cancer and normal adjacent tissues.

		**Negative**	**1%–50%**	**50%+**	***P***
ER-α	Cancers	15 (17.0%)	28 (31.8%)	45 (51.1%)	
	Adjacent Tissues	1 (1.8%)	48 (84.2%)	8 (14.0%)	0.001
ER-β	Cancers	2 (2.3%)	43 (48.9%)	43 (48.9%)	
	Adjacent Tissues	3 (5.3%)	21 (36.8%)	33 (57.9%)	N.S.
PR-A	Cancers	50 (56.8%)	31 (35.2%)	7 (8.0%)	
	Adjacent Tissues	25 (43.9%)	28 (49.1%)	4 (7.0%)	N.S.
PR-B	Cancers	45 (51.1%)	29 (33.0%)	14 (15.9%)	
	Adjacent Tissues	26 (45.6%)	20 (35.1%)	11 (19.3%)	N.S.
CDC47	Cancers	1 (1.1%)	64 (72.7%)	23 (26.1%)	
	Adjacent Tissues	0 (0.0%)	57 (100%)	0 (0.0%)	0.001

**Table 4A.** Correlation between STS immunoreactivity and clinical parameters in 88 breast carcinomas.

		**STS immunoreactivity^†^**		***P***
		**1%–50% (n=31)**	**50%+ (n = 57)**	
Type*	Infiltrative ductal	28 (99.3%)	50 (87.7%)	N.S.
	Infiltrative lobular	1 (3.3%)	3 (5.3%)	
	Others	1 (3.3%)	4 (7.0%)	
Stage*	I	1 (3.2%)	6 (10.5%)	N.S.
	II	23 (74.2%)	45 (78.9%)	
	III	6 (19.4%)	6 (10.5%)	
Age*	Years (average)	51.3 ± 8.3	50.8 ± 9.7	N.S.
Menopause status*^‡^	No (≤49-years-old)	14 (46.7%)	31 (54.4%)	N.S.
	Yes (≥50-years-old)	16 (53.3%)	26 (45.7%)	
ER-α	Negative	8 (25.8%)	7 (12.3%)	N.S.
	1%–50%	11 (35.5%)	17* (29.8%)	
	50%+	12 (38.7%)	33 (57.9%)	
ER-β	Negative	1 (3.2%)	1 (1.8%)	N.S.
	1%–50%	18 (58.1%)	25 (43.9%)	
	50%+	12 (38.7%)	31 (54.4%)	
PR-A	Negative	19 (61.3%)	31 (54.4%)	N.S.
	1%–50%	10 (32.3%)	21 (36.8%)	
	50%+	2 (6.5%)	5 (8.8%)	
PR-B	Negative	20 (64.5%)	25 (43.9%)	N.S.
	1%–50%	9 (29.0%)	20 (35.1%)	
	50%+	2 (6.5%)	12 (21.1%)	
CDC47	Negative	1 (3.2%)	0 (0.0%)	<0.05
	1%–50%	26 (83.9%)	38 (66.7%)	
	50%+	4 (12.9%)	19 (33.3%)	
EST1E1	Negative	4 (12.9%)	1 (1.75%)	0.064
	1%–50%	14 (45.2%)	16 (28.1%)	
	50%+	13 (41.9%)	40 (70.2%)	

* Histological type, tumor stage and age at surgery were unknown for one patient.

† No cancer tissues were negative for STS; that’s why there is no «negative» column.

‡ Menopausal status was unknown for all patients. It was assume that 50-year-old patients and older were menopaused while 49-year-old and younger were not menopaused.

**Table 4B.** Correlation between EST1E1 immunoreactivity and clinical parameters in 88 breast carcinomas.

		**EST1E1 immunoreactivity**			***P***
		**Negative (n = 5)**	**1%–50% (n=30)**	**50%+ (n = 53)**	
Type*	Infiltrative ductal	4 (80.0%)	28 (93.3%)	46 (88.5%)	N.S.
	Infiltrative lobular	0 (0%)	0 (0.0%)	3 (5.8%)	
	Others	1 (20%)	2 (6.7%)	3 (5.8%)	
Stage*	I	0 (0%)	1 (3.3%)	6 (11.5%)	N.S.
	II	4 (80.0%)	26 (86.7%)	38 (73.1%)	
	III	1 (20%)	3 (10.0%)	8 (15.4%)	
Age*	Years (average)	52.4 ± 7.8	50.7 ± 8.6	51.0 ± 9.8	N.S.
Menopause status*^‡^	No (≤49-years-old)	2 (40%)	15 (50%)	28 (54%)	N.S.
	Yes (≥50-years-old)	3 (60%)	15 (50%)	24 (46%)	
ER-α	Negative	2 (40%)	5 (16.7%)	8 (15.1%)	N.S.
	1%–50%	2 (40%)	9 (30.0%)	17 (32.1%)	
	50%+	1 (20%)	16 (53.3%)	28 (52.8%)	
ER-β	Negative	1 (20%)	1 (3.3%)	0 (0.0%)	<0.005
	1%–50%	1 (20%)	21 (70%)	21 (39.6%)	
	50%+	3 (60%)	8 (26.7%)	32 (60.4%)	
PR-A	Negative	4 (80.0%)	18 (60%)	28 (52.8%)	N.S.
	1%–50%	1 (20%)	11 (36.7%)	19 (35.8%)	
	50%+	0 (0%)	1 (3.3%)	6 (11.3%)	
PR-B	Negative	3 (60%)	21 (70%)	21 (39.6%)	<0.05
	1%–50%	2 (40%)	7 (23.3%)	20 (37.8%)	
	50%+	0 (0%)	2 (6.7%)	12 (22.6%)	
CDC47	Negative	1 (20%)	0 (0.0%)	0 (0.0%)	<0.05
	1%–50%	4 (80.0%)	26 (86.7%)	34 (64.2%)	
	50%+	0 (0%)	4 (13.3%)	19 (35.8%)	
STS	Negative	0 (0%)	0 (0%)	0 (0%)	0.064
	1%–50%	4 (80.0%)	14 (46.7%)	13 (24.5%)	
	50%+	1 (20%)	13 (53.3%)	40 (75.5%)	

* Histological type, tumor stage and age at surgery were unknown for one patient.

‡ Menopausal status was unknown for all patients. It was assume that 50-year-old patients and older were menopaused while 49-year-old and younger were not menopaused.

## List of Abbreviations

Δ4-dione: Androstenedione;
17β-HSD: 17β-hydroxysteroid dehydrogenases;
CDC47: cell division control protein 47;
DHEA: dehydroepiandrosterone;
DHEA-S: dehydroepiandrosterone sulfate;
E1: estrone;
E1-S: estrone sulfate;
E2: estradiol;
E2-S: estradiol sulfate;
ER-α: estrogen receptor α;
ER-β: estrogen receptor β;
EST1E1: estrogen sulfotransferase 1E1;
PBS: phosphate-buffered saline;
PR-A: progesterone receptor A;
PR-B: progesterone receptor B;
STS: steroid sulfatase.
